# SARS-CoV-2 Infection and Racial Disparities in Children: Protective Mechanisms and Severe Complications Related to MIS-C

**DOI:** 10.1007/s40615-021-01092-7

**Published:** 2021-07-13

**Authors:** Sanjana Kurup, Regan Burgess, Fatou Tine, Ann Chahroudi, Dexter L. Lee

**Affiliations:** 1 Department of Physiology and Biophysics, Howard University College of Medicine, 520 W Street, NW, Washington, DC 20059 USA; 2 Department of Pediatrics, Emory University School of Medicine, Atlanta, GA 30322 USA; 3 Center for Childhood Infections and Vaccines of Children’s Healthcare of Atlanta and Emory University, Atlanta, GA 30322 USA

**Keywords:** SARS-CoV-2, MIS-C, Kawasaki Disease, Pediatrics, Health disparities

## Abstract

A novel coronavirus has resulted in a pandemic with over 176 million confirmed cases and over 3.8 million recorded deaths. In the USA, SARS-CoV-2 infection has a significant burden on minority communities, especially Hispanic and Black communities, which are overrepresented in cases compared to their percentage in the population. SARS-CoV-2 infection can manifest differently in children and adults, with children tending to have less severe disease. A review of current literature was performed to identify the hypothesized protective immune mechanisms in children, and to describe the rare complication of multisystem inflammatory syndrome in children (MIS-C) that has been documented in children post-SARS-CoV-2 infection. Epidemiologic data and case studies have indicated that children are less susceptible to more severe clinical features of SARS-CoV-2 infection, a finding that may be due to differences in the cytokine response generated by the innate immune system, high amounts of ACE-2 which maintain homeostatic functions by preventing inflammation, and trained immunity acquired from regular vaccinations. Despite these protective mechanisms, children are still susceptible to severe complications, such as MIS-C. The racial disparities seen in MIS-C are extremely apparent, and certain populations are more affected. Most specifically, 33% of MIS-C patients are Hispanic/Latino, and 30% Black. Current studies published on MIS-C do not detail whether certain symptoms are more present in certain racial/ethnic groups. Knowledge of these disparities could assist health care professionals with devising appropriate strategies for post-acute SARS-CoV-2 infection follow-up in children as well as vaccine distribution in specific communities to help slow the spread of SARS-CoV-2 infection, and ultimately reduce the potential for complications such as MIS-C.

## Background

In 2019, a novel CoV-strain (SARS-CoV-2) originated as a pneumonia outbreak in Wuhan, China, and is the cause of the current global pandemic [1]. Since the initial outbreak, SARS-Cov-2 has spread internationally, with more than 176 million confirmed cases and over 3.8 million recorded deaths [2]. In the USA, there have been more than 33.5 million confirmed cases and over 600,000 deaths [3]. SARS-CoV-2 belongs to the beta-coronavirus family, a large group of enveloped, single-stranded zoonotic RNA viruses. The symptoms experienced by individuals infected with SARS-CoV-2 are wide-ranging from benign to more severe complications. Affected systems include the respiratory, gastrointestinal, and cardiovascular systems causing symptoms such as bronchitis, pneumonia, multi-organ failure, and death [1]. Severe acute respiratory distress syndrome seen in SARS-CoV-2 infection presents with edema, hemorrhage, intra-alveolar fibrin deposition, and vascular changes characterized by thrombosis formation, micro-angiopathy, and thrombosis mediated by pro-inflammatory cytokines [4].

Children appear to be less affected by SARS-CoV-2 infection than adults [5, 6]. Although clinical manifestations of SARS-CoV-2 are mostly reported to be mild in children, a new manifestation of SARS-CoV-2 infection has been identified in pediatric patients is a Kawasaki Disease-like condition, known as multi-system inflammatory syndrome in children (MIS-C). MIS-C is a severe hyperinflammatory illness seen in children and adolescents that typically occurs 2–6 weeks after acute SARS-CoV-2 infection [7]. Unlike Kawasaki Disease, which is much more common in Asia compared to Europe and America, MIS-C has been demonstrated to affect particularly Black and Hispanic children [8–11]. In the USA, there have been 3742 cases of MIS-C and 35 deaths reported in 48 states and 3 additional US jurisdictions according to the Centers of Disease Control and Prevention [12].

This review will aim to summarize the known and hypothesized mechanisms which protect children from severe symptoms following SARS-CoV-2 infection, identify similarities and differences between Kawasaki Disease and MIS-C, and describe the racial/ethnic disparities seen in severe inflammatory complications such as MIS-C. A description of the racial and ethnic disparities within the population affected by MIS-C is particularly important and could help provide a strategy for vaccine distribution within the younger population. Moreover, it could also assist healthcare professionals with a more focused follow-up of care in the targeted population.

## Protective Mechanisms in Pediatric Patients

In children, symptoms of SARS-CoV-2 are often asymptomatic or may resemble a typical upper respiratory infection [13]. Due to lower respiratory tract infection being less common, chest pain/discomfort and dyspnea are not typically seen in children [13]. However, lethargy, myalgia, nasal congestion, and sore throat are observed more often in children compared to adults [13]. Both the adaptive and innate immune systems are important to combat SARS-CoV-2 infection and have a direct impact on how children may elicit a different immune response and thus a different clinical course [14]. There are a few proposed mechanisms on why children are less affected by severe SARS-CoV-2 infection and they involve the relationship of ACE-2 and SARS-CoV-2 binding, the immune response to a high viral load, and trained immunity.

## ACE-2 Expression

The angiotensin converting enzyme-2 (ACE-2) is considered to be the target receptor for SARS-CoV-2 in humans, and as such, the renin-angiotensin-aldosterone system has become one of the pathways studied in order to understand the pathogenesis of SARS-CoV-2 infection [15]. ACE-2 is present in many organs, but most specifically the lungs and blood vessels. The main function of the enzyme is to reduce angiotensin II (Ang II) which functions to increase blood pressure and inflammatory processes, so ultimately the enzyme helps return the body to homeostasis [15]. During a SARS-CoV-2 infection, ACE-2 is directly occupied through binding to the SARS-CoV-2 spike glycoprotein receptor binding domain (RBD) and its expression is downregulated. As a result, less ACE-2 is available to decrease Ang II levels [15]. Ang II levels have shown to be extremely elevated in COVID-19 patients and other severe respiratory diseases [15]. Ultimately, the increased levels of Ang II correlate to more severe injury of the lungs, further perpetuating respiratory distress [16]. Angiotensin converting enzyme-2 (ACE-2) decreases with age and other common comorbidities such as hypertension and diabetes [15, 17]. Thus, children possessing high ACE-2 activity may act as a protective mechanism against a more severe immune response from SARS-CoV-2 infection [15]. In addition, recent studies have suggested ACE-2 levels vary depending on location in the body. For example, it has been shown that children have lower expression of ACE-2 in the nasal epithelium, while having higher expression of ACE-2 in the lower respiratory tract [15, 18]. Decreased ACE-2 in nasal epithelium decreases the ability of SARS-CoV-2 to bind and establish infection, while increased ACE-2 in the lower respiratory tract allows unbound ACE-2 to maintain homeostasis against SARS-CoV-2 binding [18]. Both of these mechanisms suggest that children would be less infected and, if infected, would manifest less severe symptoms.

## Cytokine Response

The pediatric immune system responds to a high viral load much differently than adults. When a high viral load is present in adults, the innate immune system will respond excessively and generate a cytokine storm, a pro-inflammatory response that may cause damage to cells, tissues, and organs in response to an acute SARS-CoV-2 viral infection [14]. The excessive immune response caused by the cytokine storm perpetuates further destruction of cells, tissues, and organs. Most specifically, the major distinct cytokines associated with adult presentations of SARS-CoV-2 were found to be IL-7 and IL-8, which were not present in pediatric cases [19]. IL-8, a chemoattractant cytokine for neutrophils, has been indicated to cause lymphopenia with SARS-CoV-2 infection, while high levels of IL-7 have been shown to maintain T cell counts to fight infection [19]. Children do not exhibit the same pattern of cytokine response as seen in adults, which may act as a protective mechanism in developing a more severe clinical response to SARS-CoV-2 infection [14].

## Trained Immunity

Another proposed theory why children are less affected by infection is the idea of trained immunity. Trained immunity is the long-term reprogramming of innate immune cells to provide a more beneficial response to pathogens and is often due to epigenetic changes, previous viral infection, and vaccination [15]. While cells of the innate immune system are not typically characterized by being able to provide a “memory” response to specific pathogens as seen in the adaptive immune system, there is now increased evidence that the innate immune system can provide some semblance of an adaptive response to a nonspecific pathogen. Trained immunity specifically leads to an altered secondary response when innate immune cells encounter a pathogen that was not seen in the primary response [20]. The altered secondary response is typically a short-term effect and will not show prolonged memory. For example, a recent study conducted by the New York Institute of Technology (NYIT) compared SARS-CoV-2 infection rates in various countries and its correlation to the Bacille Calmette-Guerin (BCG) childhood vaccination. The BCG vaccination is often given to children to prevent tuberculosis infection but can also provide protection against other respiratory infections. The study suggests that countries without a universal policy for BCG vaccination (such as the USA) were much more severely affected by SARS-CoV-2 [21]. Other countries with strict policies of BCG vaccination were found to have much lower severity and rates of SARS-CoV-2 infection, although the arrival of new strains may not show the same relationship with vaccination and outcomes [21 ].Trained immunity, due to regular vaccine inoculation or frequent viral infections from other common cold coronaviruses, may provide protection from SARS-CoV-2 and may also provide a potential explanation for the reduced severity of SARS-CoV-2 in children [15]. This may be especially important as vaccines for SARS-CoV-2 have become available, and it has yet to be established how immunity from vaccination may further protect children from severe complications.

## Complications of SARS-CoV-2 in Pediatric Patients

Although children are more protected from developing serious infection with SARS-CoV-2, over 15,000 children have been hospitalized with COVID-19 in the USA, with 306 deaths as of 5/06/2021 [22]. Further, some children develop a rare, severe complication of SARS-CoV-2 infection known as multisystem inflammatory syndrome in children (MIS-C), which has some features that are similar to Kawasaki disease (KD) [23]. KD, also known as mucocutaneous lymph node syndrome, is the most common primary vasculitis seen in pediatric patients [23]. It is a self-limiting disease that presents with three phases consisting of fever, rash, gastrointestinal symptoms, and complications including coronary artery aneurysm and shock [23, 24]. The cause of Kawasaki disease remains unclear, but it is suspected to be associated with production of self-reactive antibodies following a viral infection affecting mucosal surfaces and involving IgA-producing plasma cells [19]. Several viral agents have been implicated in this disease, including seasonal coronaviruses [23].

An inflammatory condition similar to Kawasaki disease has been reported within the pediatric population as a complication of COVID-19. This condition has been cited in the USA as pediatric multisystem inflammatory syndrome in children (MIS-C) and in Europe as paediatric inflammatory multisystem syndrome temporally associated with SARS-CoV-2 infection (PIMS-TS) [23]. The CDC describes cases of MIS-C as occurring in children less than 21 years of age who have either tested positive for current or recent SARS-CoV-2 infection via RT-PCR, serology, or antigen test, or had known exposure to a COVID-19 case within the past 4 weeks of their onset of symptoms. The child must present with a fever ≥ 38.0 degrees Celsius for 24 h or more, laboratory evidence of inflammation, and severe illness requiring hospitalization with involvement of at least two organ systems. Once all other possible causes are ruled out, including sepsis and toxic shock syndrome, a diagnosis of MIS-C is made [25]. Similarities between MIS-C and KD include rash, elevated inflammatory biomarkers, redness on the tongue known as “strawberry tongue”, and lymphadenopathy. A “strawberry tongue” is a red bumpy tongue with enlarged taste buds are symptoms of underlying conditions like KD. Once the disease is treated, the tongue will return back to its normal color and appearance. However, MIS-C seems to differ from KD by affecting older patients, having greater gastrointestinal involvement, and being more likely to cause left ventricular systolic dysfunction [21, 26]. MIS-C also presents some key differences in immunologic mediators compared to KD. Lower naive T cell (mostly CD8+ T cells) populations have been reported in MIS-C, whereas lower memory terminally differentiated effector CD4+ T-cells have been reported for KD [19]. Although the exact mechanism for this is unclear, this is congruent with several studies that have reported defective T-cell responses in adults with severe disease as a result of COVID-19 [19]. In addition, KD has also been shown to predominantly affect Asian pediatric populations, while MIS-C typically affects Black and Hispanic pediatric populations [27].

## Racial Disparities of MIS-C

Many studies published on COVID-19 have highlighted the racial/ethnic and socioeconomic disparities in rates of infection of SARS-CoV-2 in adults. A study conducted in Washington, D.C., aimed to examine whether similar disparities were present in children with SARS-CoV-2 infection [28]. A planned cross-sectional analysis was performed at an urban SARS-CoV-2 testing site that specifically serviced pediatric patients. One thousand children were tested between 03/21/2020 and 04/28/2020, and 207 positive cases were reported. Among these positive cases, Hispanic children represented 46.4% of cases and Non-Hispanic, Black children represented 30.0% of cases [28]. Non-Hispanic, White children represented 7.3% of positive cases [28]. This can be compared to the DC population with 11.3% being Hispanic, 46% being Non-Hispanic Black, and 46% being Non-Hispanic White [29]**.** These findings suggest that SARS-CoV-2 impacts children of marginalized populations at higher rates than children who come from racial/ethnic majority groups and higher socioeconomic status (SES), similar to how COVID-19 affects adults [28]. Recent studies have shown children are much more likely to acquire infection from an adult close contact, rather than from other children [30]. Potential factors driving this disparity include larger household size, essential worker parents, and use of public transportation that may all increase exposure risk for children. These factors, which are more common among minority groups, predispose these populations to contracting SARS-CoV-2, and spreading the virus to their children.

As described above, MIS-C is a rare but potentially life-threatening complication of SARS-CoV-2 infection in children. Thus, the factors influencing SARS-CoV-2 infection also play a role in which children develop MIS-C. However, the specific set of genetic, immunologic, and environmental triggers for MIS-C is unknown. Keeping this in mind, this review delves deeper into COVID-19-related racial disparities by examining trends in presentation of MIS-C.

The CDC website currently reports 3742 national cases of MIS-C and 35 total deaths [12]. Of the data reported to the CDC, 1166 of those cases have been in Hispanic or Latino children, and 1042 have been in Black, Non-Hispanic children, making at least 60% of all cases of MIS-C present in minority communities [12]. Several studies have highlighted this disproportionate racial focus of MIS-C. In New York City (NYC), where the US COVID-19 outbreak started, MIS-C cases were identified in the Spring of 2020 and on May 13, 2020, the city required mandated reporting of such cases [9, 10, 31]. Supporting these studies, a retrospective multi-institutional case study in NYC studied 33 children presenting with MIS-C and found that 45% of patients were Hispanic/Latino and 39% were Black [32]. In another cohort study examining 223 cases of MIS-C, race and ethnicity information was available for 184 patients. Compared to the percentage in the overall NYC population (22.2%) and to the percentage of hospitalized children with COVID-19 (19.9%), Black children represented a disproportionately higher percentage of MIS-C cases (34.4%) [33]. Hispanic pediatric patients, the largest group impacted by MIS-C, presented in percentages similar to NYC population. White, Asian, and Pacific-Islander children reported lower rates of MIS-C compared to their population size in NYC. Of note, 22.4% of children in this study presented with at least one comorbidity, and the two most common underlying conditions were asthma and obesity [33]. Several studies have explored the role socioeconomic status (SES) has in leading to a higher prevalence of asthma and obesity among children living in poverty, of which Black and Hispanic populations are disproportionately represented [34]. Although the development of MIS-C is most likely multifactorial as is the development of asthma and obesity, the contributing factor of SES must not be overlooked [34]. Recent research conducted at three academic centers in Massachusetts aimed to study this by examining the impact of SES and neighborhood social vulnerability index (SVI) on MIS-C development. This retrospective case-control study was conducted on 44 patients with MIS-C, of whom 44% were Hispanic and 26% were Black. Conclusions revealed that a higher SVI, lower socioeconomic status (SES), Hispanic ethnicity, and Black race were all independently associated with a higher risk of developing MIS-C [35].

There have also been many studies reported in the literature examining the various manifestations of MIS-C from outside of the USA. From these studies, additional conclusions can be drawn regarding the overrepresented racial groups in MIS-C. In France, the link between paediatric inflammatory multisystem syndrome temporally associated with SARS-CoV-2 infection (PIMS-TS) and SARS-CoV-2 infection was initially described in twenty-one patients, 18 years of age and younger, who were admitted to a university hospital with KD-like features [23]. Fifty-seven percent (n=12) of patients were of African descent. Of the major complications associated with MIS-C/PIMS-TS, 57% (n=12) presented with KD shock syndrome and 76% (n=16) with myocarditis; however, racial disparities in terms of which populations were more likely to develop severe complications were not reported. In another European study, 15 patients with PIMS-TS were studied, all of whom were of African/Afro-Caribbean, South Asian, mixed, or other minority ethnic groups [36]. This is disproportionate to the ethnic demographics of children in the West Midlands county in England, which is 3.3% black and 10% Asian [36]. Cardiac involvement was thoroughly examined, with 80% (n=12) of cases having reduced left ventricular ejection fraction, 53% (n=8) of cases with fractional shortening, and 93% (n=14) of cases with coronary artery abnormalities [36], highlighting the severity of MIS-C/PIMS-TS and need for long-term follow-up of these patients.

## Discussion

The review of the world literature has highlighted the nature of SARS-CoV-2 infection and how it manifests in children. Children are both more protected from developing symptomatic primary infection, while also displaying unique complications of infection. The protective mechanisms seen in children which cause them to experience SARS-CoV-2 infection at a lower rate and severity than adults are hypothesized to include differential ACE-2 expression, the timing and nature of the induced cytokine response, as well as routine vaccinations and frequent viral infections contributing to trained immunity.

While the literature supports the presence of immuno-protective mechanisms in children against infection with SARS-CoV-2, MIS-C is a life-threatening complication that has led to many investigations into the host factors that may be responsible for triggering this rare disease. Environmental factors have been less well-studied but given the notable racial and ethnic disparities in COVID-19 and MIS-C described above, understanding the extrinsic factors that may contribute to exposure risk and severe outcomes is important. The scarcity of data regarding outcomes is particularly striking considering the established association between race/ethnicity and comorbidities. Comorbidities such as asthma and diabetes, which disproportionately affect African-Americans and Hispanics, may contribute to a higher risk of pulmonary infections [34]. The literature has associated these comorbidities with lower socio-economic status based on air pollution, food deserts, and access to healthcare [34].

It is evident that the pathogenesis and continuing impact of SARS-CoV-2 in children has still yet to be fully understood. In all the studies described, minority populations were disproportionately more affected by MIS-C. The reported cases show that more than 60% of the affected children were from a Hispanic/Latino and Non-Hispanic Black background [12]. However, most studies have not described the correlation between racial background and susceptibility to MIS-C associated with pre-existing conditions. Majority of the studies did not report any comorbidities associated with MIS-C [37]. However, one early report showed that, among patients with pre-existing conditions, 10–39% were obese and 5–18% have a history of asthma [9]. More large-scale studies with multi-institutional data are needed to further examine the differential presentations of MIS-C. This is essential to determine if there is a link between certain comorbidities and the development of MIS-C, especially in vulnerable racial/ethnic groups. There are many factors affecting vulnerable racial/ethnic groups: (1) the number of people per home, (2) essential jobs that do not allow the flexibility of working from home, (3) a greater prevalence of underlying medical conditions, and (4) reduced access to health care have been implicated in the greater incidence of SARS-CoV-2. Knowledge of these factors can help to identify disparities, and ultimately aid in finding ways to minimize or combat complications in vulnerable populations.

Lastly, the continuous rise of COVID-19 cases has required the urgent need to develop vaccines to slow the spread of the virus. Pfizer and Moderna, pharmaceutical and biotechnology companies, initially made a modified mRNA vaccine available that was modeled against the SARS-CoV-2 spike protein [38]. Johnson & Johnson has also newly released a vaccine. Even though the vaccines have not undergone the entire approval process with the FDA, they were granted an emergency use authorization to begin vaccine distribution [39]. This was based on the randomized placebo control studies that indicated the effectiveness of the Pfizer and Moderna vaccine, 95% and 94.1% respectively [39, 40]**.** Recently, SARS-CoV-2 vaccines have now become authorized for distribution for children who are over the age of 12. It is unsure how parents will feel about vaccinating their children, as there may be some hesitancy surrounding long-term benefits and risks associated with the vaccine, which may be even more pronounced in marginalized populations. Additionally, there may even be reluctance from health care providers to administer the vaccine to children who have a history of MIS-C due to risk of recurrence. If providers and parents choose not to vaccinate children with prior MIS-C and those children are Black/Hispanic, this could potentially contribute to small pockets of children not being protected against reinfection, even though they may have some protective immunity from natural infection. Further studies are needed to study the effects of the vaccine seen in children with a past history of complications from SARS-CoV-2, such as MIS-C. When specific recommendations for vaccine distribution among the pediatric population are made, it will be of the utmost importance to prioritize children with comorbidities and vulnerable racial/ethnic status.

In conclusion, additional data on the racial disparities observed in MIS-C patients may assist health care professionals with more effective treatments for post-acute SARS-CoV-2 infection follow-up in children who are diabetic, obese, asthmatic, and from Hispanic and Black backgrounds and may also reduce subsequent complications. More studies are needed to determine if children from Hispanic and Black backgrounds have differences in the localization of ACE-2 expression, innate immune system responses to SARS-CoV-2, and trained immunity from previous viral infections and vaccinations. More studies are also needed to determine the genetic, immunologic, and environmental triggers for MIS-C. The additional factors contributing to the racial disparities among MIS-C patients, social economic status, and social vulnerability index should also play a major role in the future specific recommendations for the SARS-CoV-2 vaccine distribution among the most vulnerable children.
